# Supplementary material for: Conditional chemoconnectomics (cCCTomics) as a strategy for efficient and conditional targeting of chemical transmission
Source: eLife. 2024 Apr 30;12:RP91927. doi: 10.7554/eLife.91927 (PMC11060718; doi:10.7554/eLife.91927)
Supplement: Figure 3—source data 3. [file elife-91927-fig3-data3.zip › Figure 3 source data3 part 2.zip!/Figure 3-Source data 3-part2/1025-13-CRISPResso_Report_ack040l3.zip!/CRISPResso_on_1025_13/1a.Read_barplot.pdf]

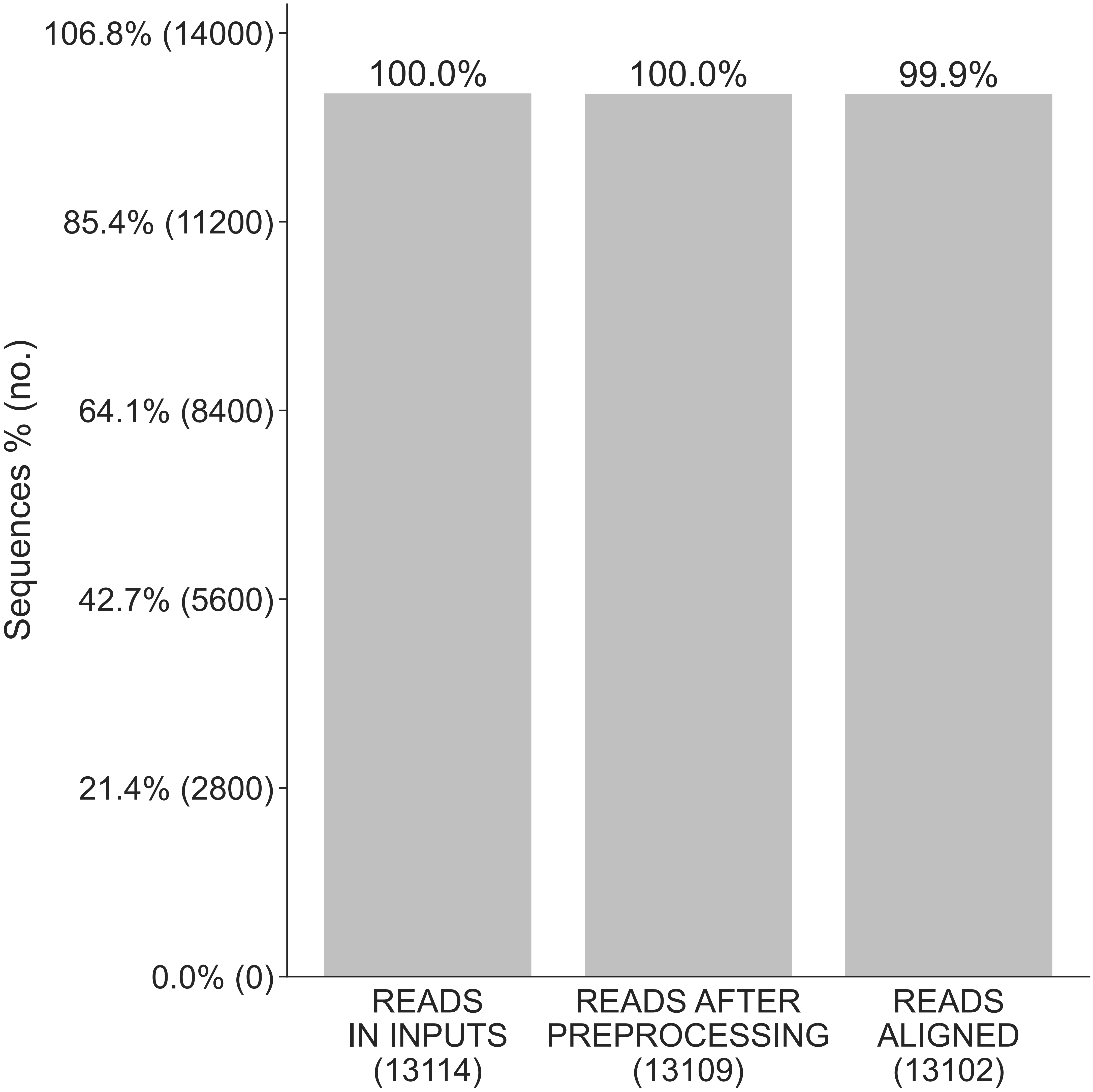

Sequences % (no.)
106.8% (14000)
85.4% (11200)
64.1% (8400)
42.7% (5600)
21.4% (2800)
0.0% (0)
100.0%
100.0%
99.9%
READS IN INPUTS (13114)
READS AFTER PREPROCESSING (13109)
READS ALIGNED (13102)
